# Primary Small Bowel Melanoma: A Case Report and Review of Literature

**DOI:** 10.3389/fsurg.2022.792243

**Published:** 2022-03-07

**Authors:** Amanda M. Graças, Willy P. Souza, Ana Carolina A. Canut, Maurice Y. Franciss, Bruno Zilberstein

**Affiliations:** ^1^Division of General Surgery, Beneficência Portuguesa of São Paulo Hospital, Gastromed-Zilberstein Institute, São Paulo, Brazil; ^2^Division of Gastrointestinal Surgery, Beneficência Portuguesa of São Paulo Hospital, Gastromed - Zilberstein Institute and São Leopoldo Mandic School of Medicine, Campinas, Brazil; ^3^Division of Gastrointestinal Surgery, Gastromed - Zilberstein Institute and São Leopoldo Mandic School of Medicine, Campinas, Brazil; ^4^Division of Gastrointestinal Surgery, Beneficência Portuguesa of São Paulo Hospital, Gastromed - Zilberstein Institute and São Leopoldo Mandic School of Medicine, Campinas, Brazil

**Keywords:** small intestine, diagnosis, melanoma, gastrointestinal metastasis, surgical treatment, case report

## Abstract

**Background:**

The present study analyzes diagnostic and therapeutic surgical aspects of primary small bowel melanoma, describing the clinical case and reviewing the literature. Malignant melanomas represent 1–3% of all malignant tumors of the gastrointestinal tract and are therefore uncommon. Only a few cases of metastatic melanoma (1–5%) are diagnosed in the early stages, while still asymptomatic. They show up as imaging exam findings and have better chance of treatment. Most of the time, the diagnosis is late and made in the presence of complications. The final diagnosis frequently depends on the surgical intervention after a serious complication.

**Case report:**

This case report refers to a 55-year-old male patient, complaining of abdominal pain, blackened stools, and palpable tumor in the abdomen for ~30 days. A tomography scan was performed, which revealed the thickening and parietal enhancement of the small intestine loops with mesenteric adenomegaly and intermingled collection. For diagnostic confirmation, a laparoscopy was performed, which revealed a tumor at the jejunal level, and its resection was performed in the same act. The anatomopathological examination revealed that it was a primary small bowel melanoma.

**Conclusion:**

The bibliographic research of the small bowel melanoma demonstrated that, in this situation, the lesion can be interpreted as a primary site or metastatic lesion, considering the possibility of a single primary lesion, whose diagnosis becomes more laborious. In such cases, adjuvant therapy must be considered. The expected 5-year survival is about 9–13%.

## Introduction

Malignant melanomas represent about 1–3% of all malignant tumors of the gastrointestinal tract (1, 2). Most of these tumors are considered secondary, metastatic lesions of a primary tumor. Both the primary and metastatic forms of intestinal melanoma are characterized by greater aggressiveness when compared with other melanoma subtypes and have a worse prognosis (1).

Only a few cases of metastatic melanoma (1–5%) are diagnosed in the early stages, while still asymptomatic (3). They show up as imaging exam findings and have better chance of treatment.

Most of the time, the diagnosis is late and made in the presence of complications, such as intussusception, acute bleeding, obstruction, and perforation. This is due, mainly, to non-specific symptoms at the beginning of the disease, and also, the available examinations are not accurate enough to determine a definitive diagnosis. In most of the cases, the final diagnosis depends on the surgical intervention, after a serious complication. Symptoms, such as weight loss, abdominal pain, constipation, hematemesis, diarrhea, anemia, fatigue, palpable abdominal mass, and blood in the stool may also be present (3).

We present a rare case of a primary small bowel melanoma, showing the challenges in its diagnosis and the importance of early surgical treatment, which can provide better quality of life and higher survival rates.

## Case Description

We present a case of a 55-year-old male that came to the Emergency Department with abdominal pain and black stools for 30 days. The decision of not looking for a medical evaluation earlier was due to the lack of other associated symptoms. He had no fever, fatigue, anorexia, diarrhea, nausea, or vomiting, but he did have marked hyporexia in the last month.

A more detailed family history of cancer was not provided. He was hypertensive, dyslipidemic, and a former smoker, and had no history of previous abdominal surgery.

On physical examination, he reported epigastrium pain. The abdomen was swollen and there was a palpable mass extending from the mesogastrium to the hypogastrium.

He was normocardic and his axillary temperature measured 36.5°C. The chest X*-*ray was normal. His oxygen saturation was monitored and remained above 97% without supplemental oxygenation. At the moment of hospitalization, the exams showed 8,000 leukocytes, C-reactive protein (CRP) was 5.1, hemoglobin was 9.8, and hematocrit was 30.2%, keeping hemodynamic stability and not showing large variations during the whole monitoring period. All serologies for infectious diseases were normal.

The investigation was proceeded by a CT of the abdomen with endovenous contrast. A confluence of small bowel loops with parietal thickening and partially defined limits, located in the infraumbilical quadrant and the left iliac fossa, was visualized. In addition, there was an unorganized collection, as well as a slight blur of mesenteric fatty planes, which suggested the presence of a regional blockade. Mesenteric adenomegaly was noted. The investigation continued by performing an upper digestive endoscopy (EDA), colonoscopy, and abdominal nuclear MRI.

**Figure 1:**
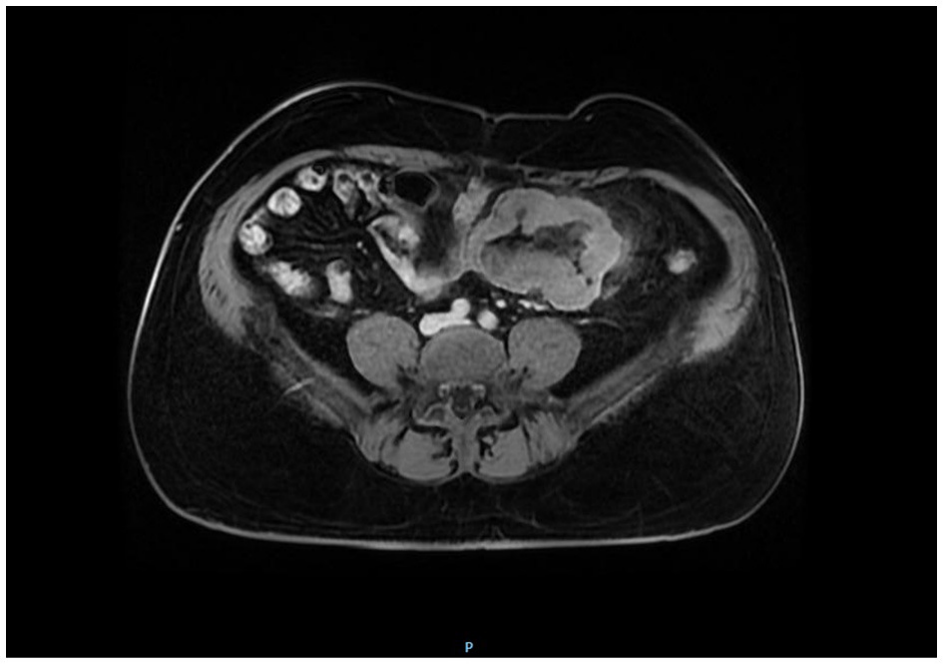
Low intensity on T1-weighted imaging. On the axial magnetic resonance image, the tumor showed an intestinal involvement due to lymphoproliferative disease.

The MRI examination showed non-obstructive and asymmetric circumferential parietal thickening of a segment of the small bowel located on the left flank, ~10.6 cm in length. Dilation of the respective lumen, was also noted, where the images suggested ulcerations. It is possible to observe the tumor on axial magnetic resonance (Figure 1), and its intestinal involvement. Lymph node enlargement up to 2.0 cm in the mesentery was visualized. Therefore, lymphoproliferative disease was considered.

The colonoscopy showed diverticulosis of the right colon and hemorrhoidal disease. An upper digestive endoscopy showed no abnormalities. Tumor markers were measured, and they were all negative. The hypothesis of adenocarcinoma of the small bowel or abdominal tuberculosis (TB) were considered. The tuberculin skin test (PPD) was suspicious, with 12 mm of skin reaction and, due to the presence of the tumor of the small bowel and enlarged mesenteric lymph nodes; there was a need to rule out the possibility of extrapulmonary TB as well.

An enteroscopy was performed, it showed mild erosive gastritis and an increased jejunal lesion, about 150 cm after the Treitz angle, with a calcified, ulcerated appearance, and a friable necrotic bottom, with signs of recent bleeding, circumferentially involving the loop to an extent of 5 cm. The anatomopathological examination of the biopsies taken from the lesion was inconclusive.

A diagnostic laparoscopy was performed. During the procedure, blocked loops adhered to the colon were seen, located 55 cm from the Treitz angle and 300 cm from the ileocecal valve. The blockage was released and the small bowel tumor was identified. Segmental enterectomy of the jejunal loops with adequate surgical margins and retroperitoneal and pelvic lymphadenectomy were performed. The reconstruction of the small bowel was performed with simple enteroanastomosis by videolaparoscopy.

As the colon was involved, the resection of the descending and sigmoid colon was performed with the reconstruction through latero-lateral anastomosis. Intraoperative pathological study was not possible.

**Figure 2:**
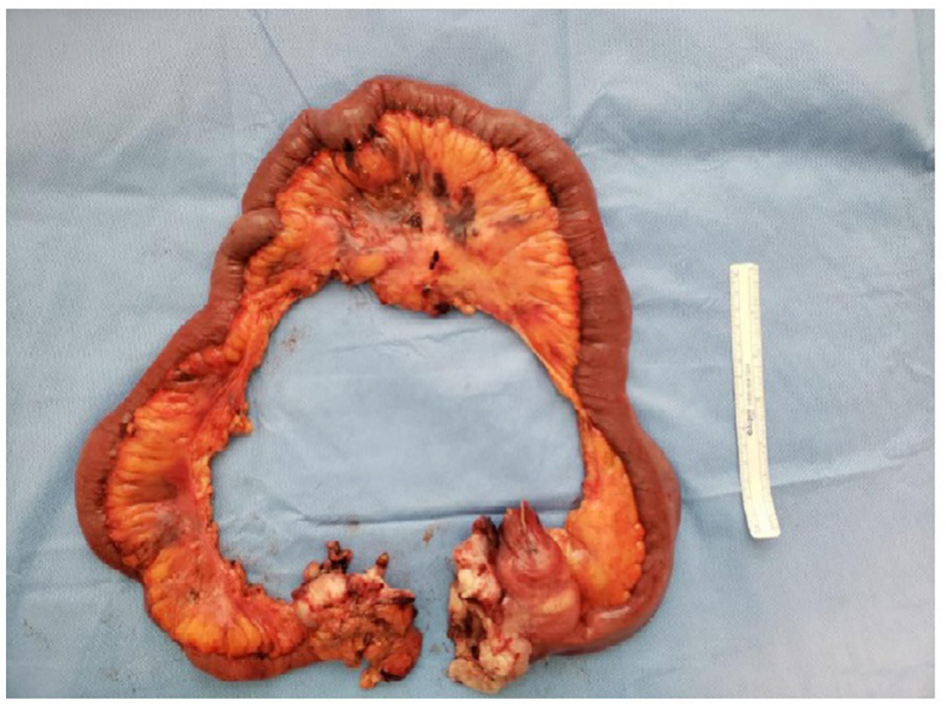
Surgical specimen. Small bowel segment. Serosa partially covered by adipose tissue. Serous tumor implants and mucosa with usual pleating.

**Figure 3:**
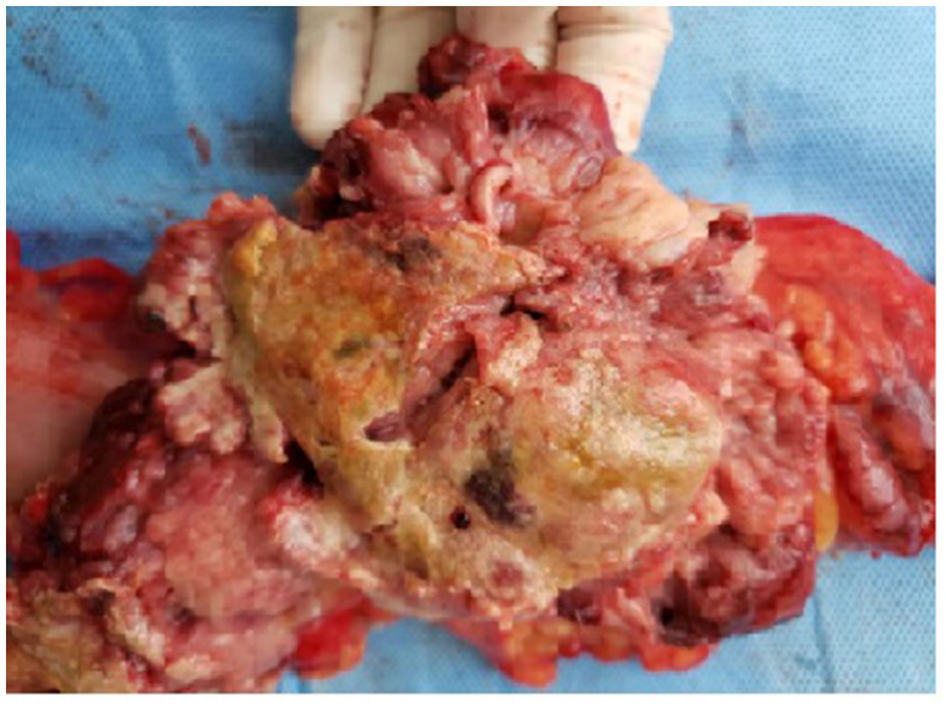
Small bowel epithelioid lesion.

**Figure 4:**
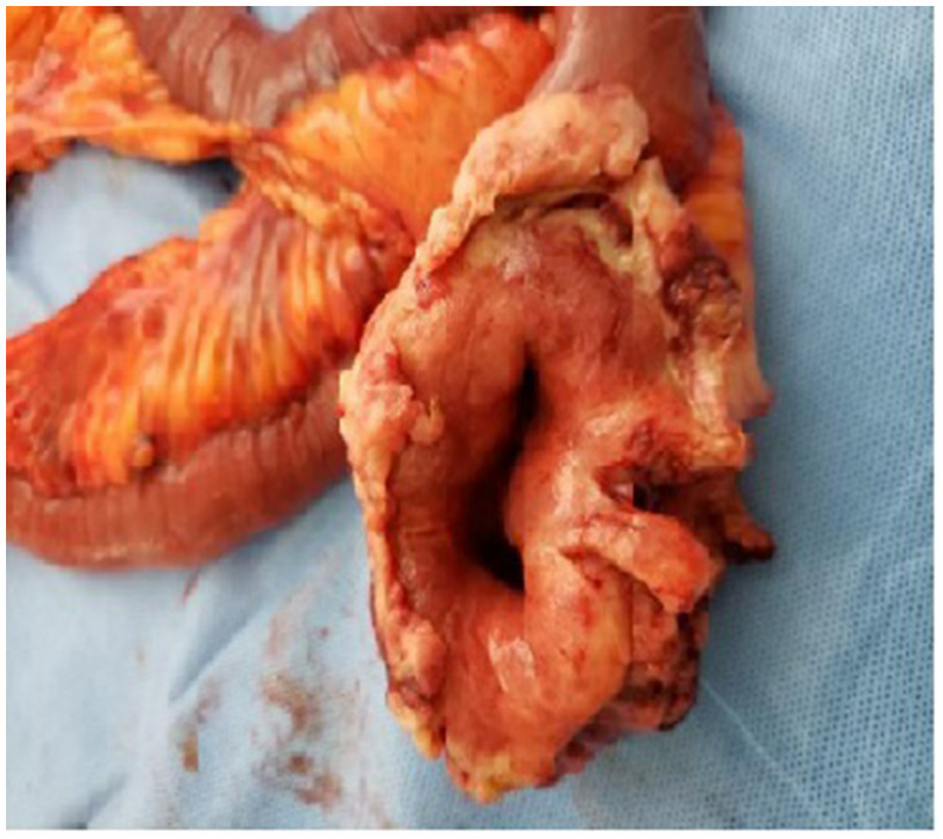
Circumferential wall thickening.

Figure 2 shows a malignant melanoma in the small bowel segment and serosa partially covered by adipose tissue. In Figure 3, the surgical specimen provides an epithelioid lesion segment found intraoperatively with jejunal perforation caused by melanoma. Figure 4 presents the circumferential wall thickening in the jejunum wall found intraoperatively.

The anatomopathological analysis of the small bowel segment and of the resected colon revealed an ulcerated sessile lesion and the presence of intestinal perforation by the tumor itself, with an extension to the adjacent adipose tissue and the formation of a tumor mass of 13.0 cm × 4.5 cm, which adhered to the large intestine. A tumor implant area measuring 5.0 cm × 4.5 cm × 3.5 cm was noted and it did not infiltrate the intestinal wall. The mucosa showed usual folding. After opening the small bowel, an ulcerated lesion with high edges was noted. When sliced, the lesion was 6.5 cm thick and had a perforation involving the surrounding adipose tissue adhered to it. There was a 13.0 cm × 4.5 cm peritoneal tumor involving the colon, which measured 17.0 cm × 3.0 cm in diameter.

The microscopic study revealed a metastatic melanoma in the jejunum and melanoma metastasis in one of the 22 regional lymph nodes dissected. Additionally, the metastatic melanoma was also observed in three pieces of adipose tissue from the pelvic lymphadenectomy.

Histological sections from the jejunal resection and left colectomy demonstrated the metastatic melanoma and traces of intense and diffuse expression of S100-protein and Melan A in the immunohistochemical analysis.

The patient had a good postoperative evolution and, when the diagnosis of small bowel melanoma was defined, he was referred for outpatient follow-up to start cancer treatment.

After confirming the diagnosis of metastatic melanoma, no evidence of a previous cutaneous lesion on a repeated physical examination was found, and also, there were no other gastrointestinal lesions. Therefore, the conclusion was a primary small bowel melanoma.

After 3 months, a control CT scan showed an increase in the dimensions of mesenteric lymph nodes of the left flank, with signs of necrosis in between. An expansive, infiltrated, and lobulated mass was observed in the hypogastric, involving tributary branches of the superior mesenteric vein. These findings are probably related to a secondary neoplastic process, which leads to the conclusion that it is a T4 N1M1 tumor. A PET-CT exam showed fat obliteration and thickening of the peritoneal planes in the left flank and iliac fossa, associated with diffuse 18F-Fluordeoxyglucose (FDG) uptake. Mesenteric lymph nodes were observed in the region of the left flank and in the right axillary region, with radiopharmaceutical uptake suggestive of secondary neoplastic involvement.

After a few months, another PET-CT showed an increase in the dimensions of the mesenteric lymph nodes in the left flank, and the formation of an expansive mass, with irregular contours, heterogeneous enhancement, and hypodense necrotic center in contact with an enteric segment. A nodular tumor was noted in the left rectus abdominis muscle with FDG uptake, which suggested neoplastic tissue and signs of tumor progression.

The patient seemed to have understood the necessity of treatment and was referred for immunotherapy, but never returned for follow-up. For that reason, it was not possible to know if the patient was assisted in oncological treatment.

## Discussion

Small bowel melanoma rarely occurs as a primary lesion, but it can appear as a metastatic tumor of undetermined cutaneous melanomas. In this case, the patient had no history of previous cutaneous involvement or any other site that suggested an initial injury. In such cases, the diagnostic investigation must be extensive to define if the tumor is primary or secondary. A thorough dermatological and retinal examination must be done, as there are many cases of ocular melanoma described as the primary tumor. The diagnosis is not easy (4), since most cases are asymptomatic in their initial form and the exams are not definitive. In the referred case, despite having defined the tumor presence, the final diagnosis was not possible until surgical approach. The involvement of the small bowel varies from 35 to 70% in patients with malignant melanoma and gastrointestinal metastasis (5).

Small bowel melanoma usually occurs as a secondary site of a skin lesion. There are only a few cases of primary melanoma of the small bowel itself, as in the presented case. Therefore, it is a complex task to differentiate the metastatic melanoma from a primary gastrointestinal tract melanoma (2), but the hypothesis should never be ruled out.

The primary malignant gastrointestinal melanoma may affect different portions of the gastrointestinal system; 33% of the nasopharynx, 5.9% of the esophagus, 2.7% of the stomach, 2.3% of the small intestine, 1.4% of the gallbladder, 9% of the colon, 22% of the rectum, and 31% of the anal tract (6). About 20% of cases that associate gastrointestinal metastasis from melanoma depict gastric metastases, 58% are small intestine lesions and 22% affect the colorectal region. When melanoma emerges in the cutaneous surface, it disseminates priority to the submucosa of the small intestine, presumably by CCR9 lymphocytes, CCL25 thymus-expressed chemokine axis, and specific integrins (6, 7).

According to Clark level of the primary lesion, in the analysis of intestinal metastatic progression, it may represent <6% for level I, 6–24% for level II, and more than 70% in cases of level III (6). In endoscopy studies, it is possible to recognize the gastric metastasis from melanoma as black-pigmented ulcers, represented in mucosa tissue in the shape of diffuse black pigment, small size nodules in mucosa or submucosa, lesions with aspect of polyps, or extrinsic masses (6, 8). In non-usual situations, the lesions can present a non-pigmented aspect, mimicking other types of epithelial neoplasms or even MALT lymphomas (6).

Blecker et al. (9) suggested criteria in the case of diagnosing a primary intestinal melanoma. It includes no evidence of concurrent cutaneous lesions, the absence of extraintestinal metastatic stemming from melanoma and the presence of intramucosal lesions in the covering intestinal epithelium. According to this criteria, this type of melanoma can present more aggressive characteristics and with risks of worse prognosis. The lymph node involvement, advanced age, and failure to undertake the surgical resection can be considered as unfavorable conditions (9) that justify the standard treatment. This case corresponds to the risk factors and points to bad prognosis described in the criteria.

Tumors with distant metastases are considered stage M1, receiving subclassification M1a, for distant skin metastases, subcutaneous, or lymph node metastases; M1b for pulmonary metastases; M1c for visceral metastases; and M1d for brain metastases. Gastrointestinal tract metastases occur in about 20% of patients in stage IV (10).

The most common symptom is rectal bleeding. Perforation and intussusception are very rare and occurs in acute cases. The diagnosis, through colonoscopy and endoscopy, may fail to identify illness of the small bowel (4) and the CT has a diagnostic sensitivity of only 68% (3). CT and MRI can suggest alterations that can correspond to a neoplasm of the small bowel. MRI is not indicated routinely because some small tumors cannot be seen. However, CT can demonstrate small masses. The best exam for an accurate intestinal evaluation and to undertake a biopsy to confirm diagnosis is a colonoscopy.

In this case, an enteroscopy and a laparoscopy were performed, since no previous exams were able to define the diagnosis. Prakoso and Selby (3) proposed that the diagnosis can be made in patients with symptoms and history of melanoma using an endoscopic capsule, even if there has been a previous negative PET-CT (3).

The enteroscopy undoubtedly facilitates the diagnostic investigation. It can be better than the endoscopic capsule, since it allows tissue biopsy and marking of the surgical site for more accurate identification of the lesion. It can be used on patients with bowel obstruction, but with proper care, while an endoscopic capsule must not be done (1).

Complete gastrointestinal resection and metastasis resection, with a safety margin, are highly relevant prognostic factors related to the long-term survival (11). The definitive diagnosis of small bowel metastasis is best obtained after laparotomy or exploratory laparoscopy and resection of the intestinal lesion (12). In the surgical approach, performing a laparoscopic intervention on a patient with small bowel neoplasm creates the possibility of port-site metastasis.

Upfront surgical procedure is indicated for the most severe cases with symptoms that need urgent treatment, such as bleeding or obstructive gastrointestinal lesions. Surgical resection can increase the long-term survival (13).

In advanced cases, in which curative surgery is not possible, palliative tumor resection can be done, reducing symptoms and decreasing the chance of future complications. Metastasis treatment includes chemotherapy and immunotherapy (5).

Establishing a diagnosis and staging the tumor are essential steps. Adjuvant cancer treatment can improve the quality of life and slow the progression of the disease. Metastasis recurrence must be considered, they are often synchronous, can indicate progression of the disease, and result in the need for a second surgical intervention. The malignant melanoma has a poor prognosis and a 5-year survival rate of only 9–13% (14).

Multidisciplinary treatment, with adjuvant immunotherapy, can improve the prognosis. In cases of metastatic melanoma, systemic immunotherapy with monoclonal anti-CTLA4 ipilimumab has shown to be effective (6, 15). Immunotherapy was indicated but the patient did not return for consultation, having lost follow-up.

The follow-up includes colonoscopy, in case of new episodes of gastrointestinal bleeding, and endoscopy, in cases of gastric melanoma. More rarely, metastatic deposits have the potential of provoking chronic anemia. Endoscopic studies are part of the follow-up and may include patients with bland gastroenteric symptomatology (6, 16). PET-CT is mandatory to monitor the progress of the disease, its advanced stages, and to guide the oncological treatment. The oncologists have the possibility of choosing the systemic immunotherapy with the monoclonal anti-CTLA-4 antibody ipilimumab, which has been attested as therapeutically effective with metastatic melanoma. Ipilimumab, with or without a gp100 peptide vaccine, had a positive impact on the overall survival in patients with precedent metastatic melanoma treatment (6, 15).

The median overall survival was 10.0 months for patients who received ipilimumab plus gp100, when compared with 6.4 months in the case of patients that received gp100 individually. The time-period of 10.1 months was estimated as an overall survival with ipilimumab isolated (6, 17).

In the study with immunotherapy, after ipilimumab treatment, the patient was proposed to begin a second modality of treatment with the anti-PD-1 monoclonal antibody pembrolizumab (17).

This phase II study established a comparison between two types of doses (2 vs. 10 mg/kg every 3 weeks) of pembrolizumab vs. chemotherapy scheme (paclitaxel plus carboplatin, paclitaxel, carboplatin, dacarbazine, or oral temozolomide), monitoring patients with the clinical condition of metastatic melanoma that previously received ipilimumab as therapy. It was stated that both dosages of pembrolizumab had advantageous results compared with the chemotherapy in terms of progression-free survival (18).

Regarding the perspective of treatment, identifying the important points that should be addressed with the patient, according to the medical evaluation, and considering the severity of the condition, it is essential to emphasize the need for surgical treatment. This allows the patient to understand the importance of the surgical approach, its health conditions, and impact on the quality of life. This made it possible to recognize that the patient seemed confident about the surgical procedure, providing opportunities for clear communication about the neoplasia and its evolution prospects.

## Conclusion

This article shows a case of a primary small bowel melanoma, a rare pathology, with a tricky diagnosis, emphasizing the importance of establishing an accurate protocol for its treatment.

Early diagnosis and rapid resection surgery can provide better quality of life and longer symptom-free survival. When dealing with a metastatic melanoma of the small bowel, the possibility of other primary sites may be considered, but the possibility of a single primary lesion cannot be discarded. In the presented case, after confirming the diagnosis of metastatic melanoma in the small bowel biopsy, no evidence of a previous cutaneous lesion on a repeated physical examination was found. Additionally, there were no other gastrointestinal lesions. Therefore, the conclusion was a primary small bowel melanoma. The investigation should include imaging exams and consider enteroscopy, which allows a better study of the lesion. The surgical treatment of intestinal metastasis with a curative approach is viable only in a few situations. A surgical therapy is essential to treat the sickened portion of the small bowel and to resect any signs of lymphoproliferative disease. It allows primary diagnosis and can result in the increased patient survival. Primary intestinal melanomas have an aggressive course and worse prognosis, making follow-up necessary, as it carries a risk of recurrence. Patients with metastatic melanoma should be carefully screened for gastrointestinal symptoms and a timely colonoscopy may benefit these patients in detecting the early metastatic disease. Regarding the perspective of treatment and considering the severity of the condition, it becomes essential to emphasize the need for surgical treatment. It is a rare case that addresses surgical aspects in depth and discuss the surgical behavior with the scientific community.

## Data Availability Statement

The original contributions presented in the study are included in the article/supplementary material, further inquiries can be directed to the corresponding author.

## Ethics Statement

The studies involving human participants were reviewed and approved by Ethics Committee of São Leopoldo Mandic Faculty. The Ethics Committee waived the requirement of written informed consent for participation.

## Author Contributions

AG and BZ: conception, design, and administrative support. AG and WS: provision of study materials or patients, collection, and assembly of data. AG, WS, and BZ: data analysis and interpretation. AG, WS, AC, MF, and BZ reviewed the literature and contributed to manuscript drafting and manuscript writing. All authors issued final approval for the version to be submitted.

## Funding

This work was supported by the Beneficência Portuguesa of São Paulo Hospital. The funder had no role in study design, data collection and analysis, decision to publish, or preparation of the manuscript.

## Conflict of Interest

The authors declare that the research was conducted in the absence of any commercial or financial relationships that could be construed as a potential conflict of interest.

## Publisher's Note

All claims expressed in this article are solely those of the authors and do not necessarily represent those of their affiliated organizations, or those of the publisher, the editors and the reviewers. Any product that may be evaluated in this article, or claim that may be made by its manufacturer, is not guaranteed or endorsed by the publisher.
